# Are Structural Changes Induced by Lithium in the HIV Brain Accompanied by Changes in Functional Connectivity?

**DOI:** 10.1371/journal.pone.0139118

**Published:** 2015-10-05

**Authors:** Madalina E. Tivarus, Britta Pester, Christoph Schmidt, Thomas Lehmann, Tong Zhu, Jianhui Zhong, Lutz Leistritz, Giovanni Schifitto

**Affiliations:** 1 Department of Imaging Sciences, University of Rochester Medical Center, Rochester, New York, United States of America; 2 Institute of Medical Statistics, Computer Sciences and Documentation, Jena University Hospital, Friedrich Schiller University, Jena, Germany; 3 Department of Radiation Oncology, University of Michigan, Ann Arbor, Michigan, United States of America; 4 Department of Neurology, University of Rochester Medical Center, Rochester New York, United States of America; University of Amsterdam, NETHERLANDS

## Abstract

Lithium therapy has been shown to affect imaging measures of brain function and microstructure in human immunodeficiency virus (HIV)-infected subjects with cognitive impairment. The aim of this proof-of-concept study was to explore whether changes in brain microstructure also entail changes in functional connectivity. Functional MRI data of seven cognitively impaired HIV infected individuals enrolled in an open-label lithium study were included in the connectivity analysis. Seven regions of interest (ROI) were defined based on previously observed lithium induced microstructural changes measured by Diffusion Tensor Imaging. Generalized partial directed coherence (gPDC), based on time-variant multivariate autoregressive models, was used to quantify the degree of connectivity between the selected ROIs. Statistical analyses using a linear mixed model showed significant differences in the average node strength between pre and post lithium therapy conditions. Specifically, we found that lithium treatment in this population induced changes suggestive of increased strength in functional connectivity. Therefore, by exploiting the information about the strength of functional interactions provided by gPDC we can quantify the connectivity changes observed in relation to a given intervention. Furthermore, in conditions where the intervention is associated with clinical changes, we suggest that this methodology could enable an interpretation of such changes in the context of disease or treatment induced modulations in functional networks.

## Introduction

Infection with the human immunodeficiency virus (HIV) is associated with injury of the central nervous system (CNS) and HIV-associated neurocognitive disorders (HAND) [1, 2]. In the search for successful adjunctive treatments for this disease, previous studies have shown that lithium protects neurons against viral protein-induced cell death and virus-associated neurodegeneration [3, 4] and may improve cognitive function [5]. Neuroimaging biomarkers can reveal early changes in the brain structure and function that may predict, accompany and explain the therapeutic drug efficacy at a time when clinical responses are not measurable.

MRI studies of patients that have taken lithium have shown increased gray matter volume and density, supporting the observations that lithium causes neurogenesis in the brain [6]. A review of studies examining effects of lithium on neuroimaging findings in bipolar disorder concluded that lithium has normalizing effects on both functional and structural measures, meaning that the results in medicated subjects were more similar to those found in healthy individuals [7]. We have previously explored the potential clinical benefit of lithium in HIV patients with cognitive impairment, using a multi-imaging modality approach [8]. Our findings demonstrated that after lithium treatment the brain activation patterns in cognitively impaired HIV patients during an attention-switching task using functional MRI (fMRI) were more similar to those in our healthy control group. Similar normalizing changes were observed in CNS microstructure using diffusion tensor imaging (DTI), with several brain areas showing increased fractional anisotropy (FA) and decreased mean diffusivity (MD) after treatment with lithium [8]. These quantitative measures describe the extent (MD) and directional dependency (FA) of water diffusion and can be used to infer non-invasively underlying CNS microstructures as well as alternations of their integrity due to pathological conditions. However, conventional fMRI activation and DTI analyses do not reveal information about functional connections and their strength. Increased strength in the functional network may reflect increased efficiency, which in the context of cognitive impairment, would suggest that the intervention is beneficial and could potentially predict clinical benefit. We sought to further investigate if the changes observed separately in functional and anatomical measures were modulated by an increase in brain connectivity measures.

Several approaches have been used in computational neuroscience to address the concept of directed brain connectivity [9, 10]. A widely used concept for analyzing connectivity patterns based on fMRI acquisitions is dynamic causal modeling (DCM) introduced by Friston [11]. DCM is based on nonlinear state space models and Bayesian model comparisons; thus, they require a priori model specifications. Likewise structural equation modeling [12] requires prior assumptions about the connectivity structure since a constraining (anatomical) model has to be established. In structural equation modeling the data covariance structure is emphasized, that is, the covariance structure implied by the anatomical model is compared with the observed covariance structure of an unconstrained model. An additional, frequently used methodology is based on Granger’s concept of predictability [13], where various approaches may be summarized by the notion of Granger Causality (GC). GC characteristics are known in the time [14, 15] as well as in the frequency domain [16, 17]. Prior assumptions about the underlying connectivity structure are not necessary and multivariate extensions are straightforward. In most applications regarding brain connectivity, GC measures are constructed on the basis of multivariate autoregressive (MVAR) models. Examples of popular directed connectivity measures in the frequency domain include Directed Transfer Function (DTF) [18] and Partial Directed Coherence (PDC) [19]. Both approaches are based on the direct exploitation of the transfer function of the underlying MVAR process. At a given frequency, DTF corresponds to the proportion of inflow from a source channel to a sink channel related to all inflows of the sink channel. In contrast, PDC refers to the corresponding outflows of the source. Both measures range from zero to one and exhibit a natural intensity interpretation [17]. As previously demonstrated in [19], PDC identifies only direct flows between channels, whereas DTF is susceptible to indirect (e.g. cascaded) interactions. In the case where noticeable differences between signal variances exist, Baccalá et al. proposed to use generalized partial directed coherence (gPDC) [20], which represents an advancement of partial directed coherence as it is normalized by the variances of model residuals. MVAR-based characteristics can be extended to time-variant time series models to capture the temporal dynamics of underlying processes [21–23].

In this proof-of-concept study we aimed to combine information from structural and functional imaging data collected in a group of HIV-infected individuals, and used gPDC to determine how lithium induced changes in brain microstructure affected functional connectivity. Specifically, brain areas that showed microstructural changes following lithium treatment were used as regions of interest (ROI) in directed connectivity analysis of fMRI time series.

## Materials and Methods

### Subjects

A cohort of HIV- infected individuals with cognitive impairment (CI) was enrolled in a 10-week, open-label lithium study [8] at the University of Rochester. Participants were required to be on a stable antiretroviral regimen or off antiretroviral therapy for at least 8 weeks prior to study entry. After an initial baseline evaluation, subjects were instructed to begin taking lithium carbonate 300 mg PO bid at approximate 12-h intervals. Follow-up evaluations were conducted at 1, 2, 4, 6, and 10 weeks and included safety surveillance laboratory tests and lithium serum levels measurements. Clinical assessments performed at each visit included vital signs, updated diagnoses, signs and symptoms, the Karnofsky Performance Scale, and a pill count to assess medication compliance. Imaging data were collected at baseline and week 10. CI was defined as (a) performance at least 1.0 standard deviation below age- and education matched controls on two or more separate neuropsychological tests; and/or (b) performance at least 2.0 standard deviations below age- and education-matched controls on one or more separate neuropsychological tests. Normative data for neuropsychological test scores were the same as those used in the Dana and Northeast AIDS Dementia cohorts [24, 25]. A neurological examination was performed and plasma HIV RNA (Roche Amplicor HIV–1 Monitor Ultrasensitive Method) and CD4^**+**^/CD8^**+**^ cell counts and percentages were measured at study entry (viral load—6788.2 copies/ml; mean CD4^+^ count—329.33/mm^3^) and week 10 (viral load—4852.0 copies/ml; mean CD4^+^ count—270.0/mm^3^). A subset of 7 participants (4 male, age range 43–52, mean = 45.54) who had neuroimaging scanning performed before and after lithium treatment was used in the present study.

The study was reviewed and approved by the Institutional Review Board at the University of Rochester Medical Center and all subjects signed a written informed consent prior to undergoing study procedures.

### Neuroimaging

**Fig 1:**
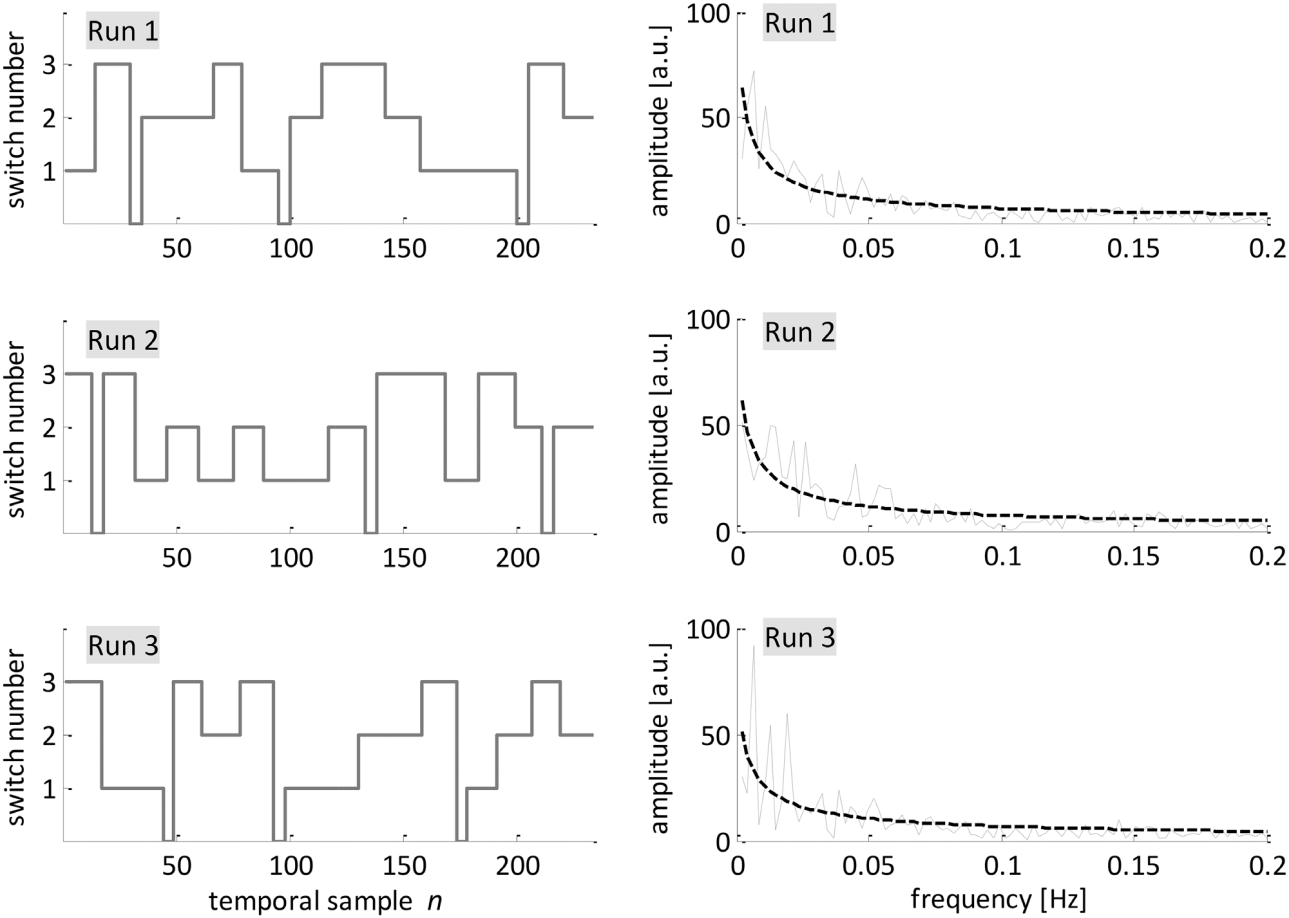
Left: Task sequence during the three experimental runs; Right: non-parametric Fourier amplitude spectra (solid lines) corresponding to experimental task sequences together with the exponential approximations (dashed lines) as used for generalized partial directed coherence aggregation.

Neuroimaging was performed before and after lithium treatment, with a period of 10 weeks between the studies. Imaging data were collected on a Siemens 3T MRI Trio system with an 8-channel head coil. High-resolution T1 weighted structural images were acquired using MPRAGE sequence (TR/TE/TI = 2530/3.39/1100 ms, FOV = 256 mm, resolution 1x1x1 mm^3^), and used for spatial normalization. DTI data were collected using a single-shot spin-echo EPI sequence with twice-refocused spin echoes (TR/TE = 10100/100 ms, resolution 2x2x2 mm^3^, iPAT (GRAPPA) acceleration factor = 2, 24 diffusion gradient directions, b = 1000 s/mm^2^ with one average, and four non-diffusion weighted images with b = 0). A series of BOLD EPI scans (GRE EPI sequence, TR/TE = 2000/30ms, resolution 4x4x4 mm^3^) were acquired while participants performed a working memory task. The task was based on Garavan et al [26] and consisted of sequences of large and small squares presented visually for 1500 ms each and intermixed with 100 ms fixation trials. Each sequence of squares was considered a condition and labeled 1-switch, 2-switch or 3-switch based on how many times the size of the squares changed during the sequence. Participants were required to retain in memory separate counts of small and large squares and report it at end of the sequence. Each imaging run was 233 time points long and consisted of 15 randomly presented conditions, five of each type: 1-switch, 2-switch and 3-switch (Fig 1 left). To avoid practice effects, the length of each of the sequences was varied between 11 to 15 squares. This ensured the number of squares presented was enough to make the task challenging, while also keeping the total run time constant. During each visit (pre or post treatment), participants performed three imaging runs, with the order of sequence presentation changing from run to run to avoid practice effects. Prior to scanning, all participants completed a brief training session to ensure comprehension of the task.

### Data pre-processing

The DTI results from a previously published study [8] were used to select the ROIs for the connectivity analysis. These were obtained using FSL [26] and SPM2 (The Wellcome Department of Imaging Neuroscience, University College London) software packages. This analysis is described in detail in our previous publication [8]. Briefly, pre-processing steps consisted of correction of eddy current distortions and motion artifacts, field-map based susceptibility artifacts correction, calculation of the diffusion tensor, and spatial normalization of tensor-derived parameter maps. Voxel-based morphometry was implemented to explore brain areas that may have been affected by lithium treatment. Due to the small data sample, an alternative to family-wise error rate based correction was adapted for multiple comparison corrections. Thus, comparisons using an uncorrected *p* = 0.001 and a cluster size threshold of 5 revealed several areas of increased FA and decreased MD (Table 1). FA increases were seen in gray matter areas including the right cerebellum, right putamen and right medial frontal gyrus whereas decreases in MD were present in the right and left frontal orbital cortex, right lateral occipital cortex and right subcallosal cortex. These areas were selected as ROIs for connectivity analysis, which was applied to fMRI data pre-processed using FSL software package. Pre-processing consisted of temporal filtering (high pass filter cutoff 264s), slice time correction, field mapping correction, and intensity normalization.

**Table 1:** Areas of significant increases in FA (A) and decreases in MD (B) from baseline to week 10 (uncorrected *p* = 0.001, cluster size threshold = 5) used as ROIs in the gPDC analysis.

FA increases		MD decreases	
MNI coordinates (x, y, z) mm	Brain region	MNI coordinates (x, y, z) mm	Brain region
(18, -67, -39)	Right cerebellum	(-14,20, -34)	Left orbital gyrus (BA47)
(20, 15, -16)	Right putamen	(16, 35, -34)	Right orbital gyrus (BA47)
(5, 53, -18)	Right medial frontal gyrus (BA10)	(60, -62, -2)	Right lateral occipital cortex (BA37)
		(12, 24, -20)	Right Subcallosal gyrus (BA11)

### Connectivity analysis

The degree of directed information transfer between nodes of the fMRI network was quantified by means of gPDC based on time-variant multivariate autoregressive (tvMVAR) processes. As a first step, the 7-dimensional fMRI time series (**y**(1),…,**y**(*N*)) were approximated by tvMVAR models

$$y(n)=\sum_{r=1}^{R}A^{r}(n)\;\cdot y(n-r)+w(n)\in {ℜ}^{D},\;n=R+1,\ldots ,N,$$ (1)

where *R* is the model order, **A** ^*r*^(*n*) (in our case *n* = 1,…,233) are real valued *D* × *D* -matrices and **w** is a *D*-dimensional white noise process (in our case *D* = 7 ROIs). The model order *R* indicates the number of samples in the past that are used to approximate the data at a certain point in time. To obtain an assessment for a suitable choice of model order, we considered prediction errors in conjunction with the number of parameters that have to be estimated, by utilizing Akaike's information criterion (AIC). AIC usually provides a reasonable initial guess. However it is known that AIC frequently overestimates the model order. That is why the initial model order was subjected to a tuning procedure ensuring a sufficient coincidence between the parametric (MVAR-related) and the Fourier power spectra, where both spectra were compared for various model orders. Finally, the smallest order was chosen, where all substantial frequency components were represented by the parametric estimation, ensuring that the parametric spectrum reconstructs all distinctive spectral components of interest. This resulted in a model order of five.

Allowing for prediction errors and complying with common experience, the tvMVAR model in fMRI analyses order was set to *R* = 5. The estimation of model parameters in Eq (1) was carried out by a time-variant, multivariate version of the Kalman filter [23]. The time courses of all 7 ROIs were integratively approximated by a 7-dimensional tvMVAR model, where every run was processed separately.

PDC is a common and well-established tool for a frequency-selective quantification of directed interaction between nodes of a multivariate process [19]. It is based on the Fourier transform of the tvMVAR model (1)

$$A(n,f)=I-\sum_{r=1}^{R}A^{r}(n)\cdot e^{-2\pi ifr}$$ (2)

with normalized frequencies *f* ∈ [0,0.5] and identity matrix **I**∈ ℜ^D×’D^. In this context, the degree of information transfer from node *j* to node *i* at time point *n* and frequency *f* can be captured by PDC, and is given by

$$\pi_{i\leftarrow j}(n,f)=\frac{\left|a_{ij}(n,f)\right|}{\sqrt{\sum_{d=1}^{D}{\left|a_{dj}(n,f)\right|}^{2}}}\in \left[0,1\right],\;i\neq j,$$ (3)

where *a*_*ij*_(*n*,*f*) denotes the (*i*,*j*)-th entry of **A**(*n*,*f*). Due to large differences in some of the estimated prediction error variances, we preferred employing generalized partial directed coherence (gPDC) which is a variance-weighted modification of PDC [20]. This scale-invariant extension of PDC allows for different signal amplitudes by weighting every model parameter in Eq (3) by the corresponding standard deviation of the MVAR prediction error at time point *n* and frequency *f* [20]. Finally, the resulting gPDC matrix is of dimensionality: (number of directed interactions) x (number of frequency bins) x (number of temporal samples). In our case it is 42×50×233 because there are 7 × 6 possible directed interactions between 7 ROIs, where each interaction is investigated for every volume (233) and 50 frequencies. Furthermore, no exogenous input function has to be introduced, as gPDC is calculated on the basis of estimated MVAR model parameters.

Due to artifacts in model residuals we chose to exclude a small number of gPDC values from further analyses. Because the model order of five implies a smearing at the transitions between different switching conditions, the first five values of a new switching condition were also rejected. Therefore, about 4% of the whole data were discarded.

A group-based quantitative analysis of functional connectivity pattern was performed in order to show an association with lithium treatment. For this purpose, a qualitative inspection of gPDC maps is not sufficient. Due to the very large time-variant frequency-selective analysis output, a suitable data reduction is necessary. Typically, advanced analyses rely on dimension reduction steps in the time and/or frequency domain before statistical models are applied [27, 28], where the specification of frequencies is hypothesis-driven. The relationship between the paradigm and the frequency information contained in *π*_i←j_(*n*,*f*) is indirectly represented by the frequency characteristics of the task-associated function describing the alternating switching blocks (Fig 1 left). To condense the large number of frequency-dependent results we aggregated gPDC values (in frequency domain) into a weighted sum, where the smoothed power spectrum of the task changes provides the weighting coefficients (Fig 1 right).

**Fig 2:**
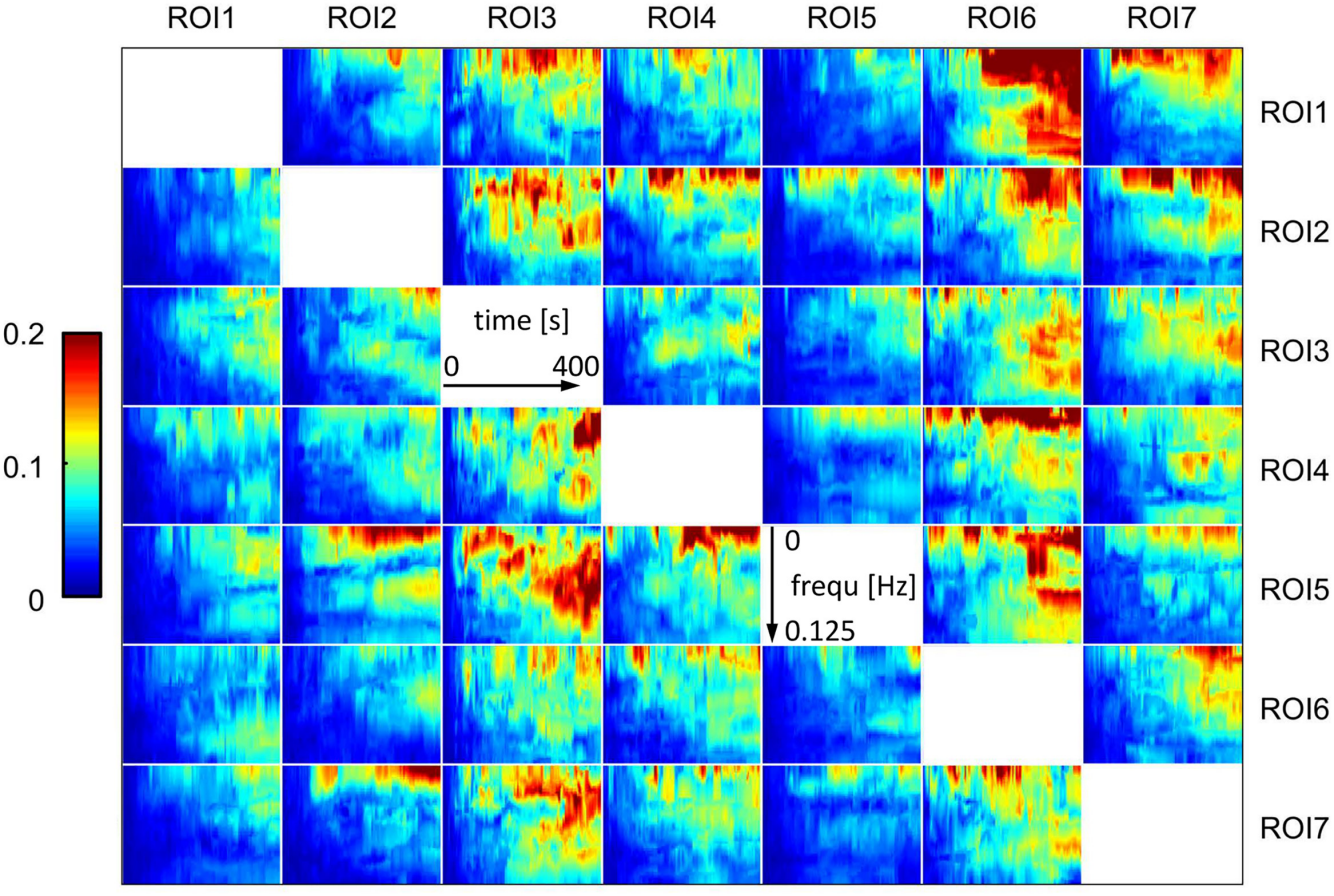
Pre lithium treatment time-variant and frequency-selective functional connectivity maps obtained by gPDC (median over all subjects, for a representative run); ROI1: Right cerebellum, ROI2: Right putamen, ROI3: Right medial frontal gyrus, ROI4: Left orbital gyrus, ROI5: Right orbital gyrus, ROI6: Right lateral occipital cortex, ROI7: Right subcallosal gyrus.

**Fig 3:**
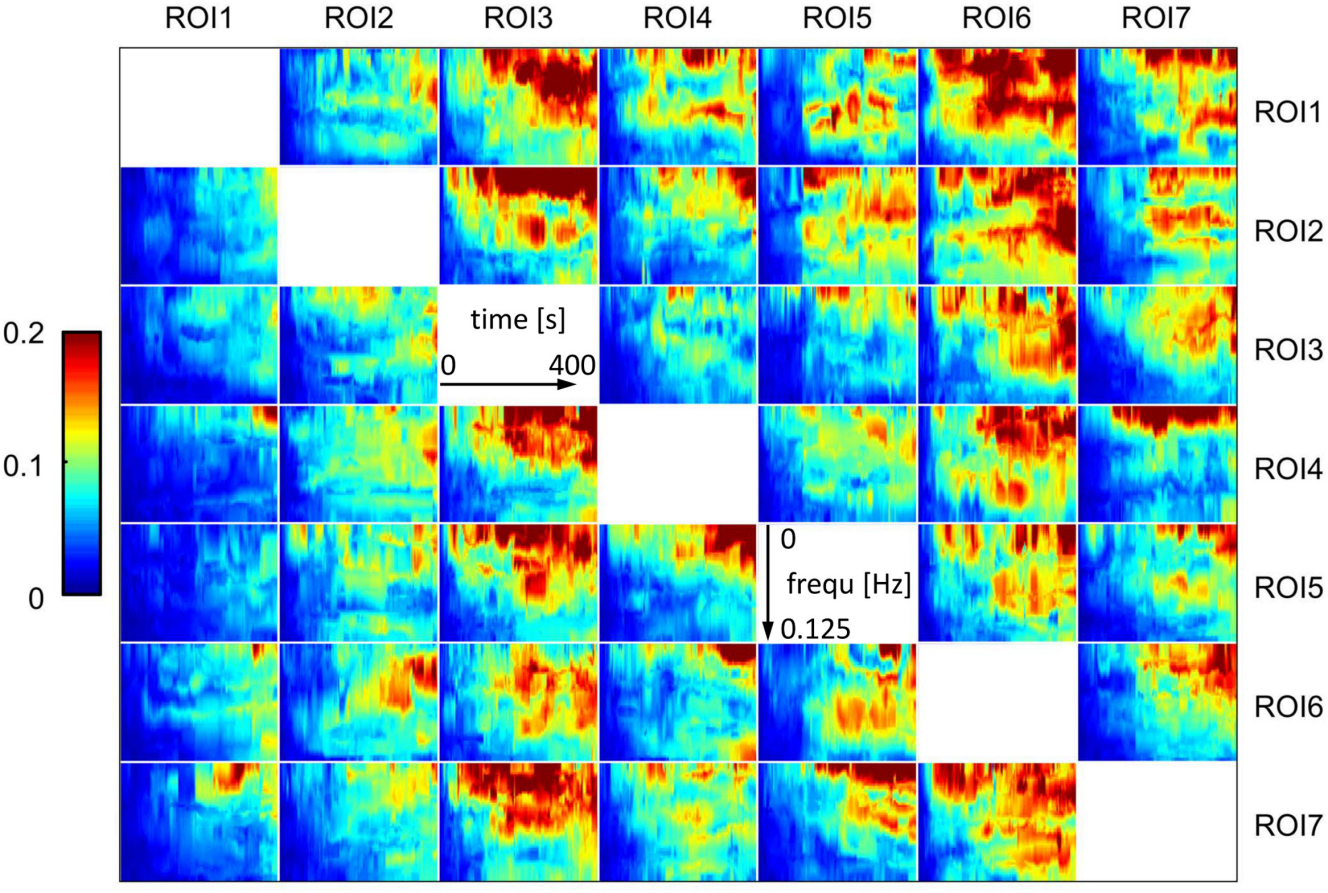
Post lithium treatment time-variant and frequency-selective functional connectivity maps obtained by gPDC (median over all subjects, for a representative run); ROI1: Right cerebellum, ROI2: Right putamen, ROI3: Right medial frontal gyrus, ROI4: Left orbital gyrus, ROI5: Right orbital gyrus, ROI6: Right lateral occipital cortex, ROI7: Right subcallosal gyrus.

To provide an overall impression of the resulting networks, median gPDC values of all subjects for pre and post lithium treatment for one representative run are presented in Figs 2 and 3. In this illustration, a time-frequency map in the *i*-th row and *j*-th column represents the time-variant, frequency-selective degree of effective connectivity from ROI *j* to ROI *i*. A qualitative comparison between Figs 2 and 3 suggests that in general gPDC values slightly increase from pre to post treatment status. However, this is merely descriptive and specific connectivity patterns are hardly identifiable due to the high amount of output data [29]. Therefore, an exhaustive evaluation of these raw results required further processing steps. This was accomplished by the already described combination of gPDC-based network measures and the subsequent statistical analysis. This detailed analysis confirmed the aforementioned descriptive assumption, namely a global lithium treatment effect on functional connectivity characteristics.

Furthermore, aggregated gPDC values of one switching condition block were condensed by applying the temporal median. Connectivity patterns obtained for any switch condition block were interpreted as a weighted complete directed graph with seven labeled nodes representing ROIs, and edge weights defined by the corresponding aggregated gPDCs. To get insights into the global connectivity structure of these networks we utilized the average node strength as a scalar characteristic. For each node, the node strength quantifies the accumulated weight of its interactions and thus measures the integration of a node with other nodes of the network. The average node strength of a network equals the averaged node strength over all nodes and measures the strength of interactions these nodes receive or transmit on average. It is a commonly used approach to analyze thresholded binary networks or series of binary networks obtained from a multiple threshold strategy [30]. In this study we focused on weighted networks in order to circumvent information loss, threshold-dependency and the complications that come from analyzing different samples of binary networks obtained from dichotomizations of the gPDC data for different, yet arbitrarily defined thresholds.

We also explored other graph indices, including the characteristic path length and weighted clustering coefficients [31–33]. The characteristic path length is the average length of shortest paths between all pairs of nodes and is related to fast and resource-efficient information transfer, assuming that information flow in the network preferably takes place on shortest connections. Clustering refers to local, segregated information processing in tightly knit neighborhoods of nodes (ROIs) and is measured as the average intensity [34] of triangles centered at each node.

In addition to the average node strength as a global network characteristic, we considered the in- and out-strength of each node (the sum of its incoming or outgoing edge weights, respectively) in order to obtain a localization of possible lithium effects. At the most detailed level of the analysis we evaluated single edge weights defined by gPDC-values.

### Statistical analysis

Since gPDC values were sampled repeatedly in switch condition blocks for each patient *v*, the correlation of these data has to be taken into account in the statistical analysis. Standard statistical methods can be applied to summarize the data (e.g. mean of all blocks); however, this is accompanied by a loss of information and statistical power. Instead, the linear mixed model (LMM) can be used to analyze efficiently a wide variety of datasets, including repeated measurements and longitudinal data [35]. Hence, a LMM with fixed and random effects was fitted to compare condensed gPDCs and derived graph indices of the different switching conditions. Fixed factors included in the model were switching conditions *S*_*sv*_, *s* = 1,2,3 and pre/post treatment status T_*tv*_, *t* = 1,2. Since we were interested in the pre/post change for each condition, we also incorporated in the model the interaction of condition and status *S*_*sv*_ * *T*_*tv*_. Measurements of each block *b* were correlated for the patients, and hence the block number *B*_*bv*_ was defined as a random effect with an appropriate AR(1) covariance structure. Finally, a random intercept *B*_0v_ was included for the patients to account for the between-subjects variation. The LMM for any dependent variable *X* is defined as

$$X_{bvst}=\beta_{0}+\beta_{0v}+\beta_{1b}B_{bv}+\beta_{1s}S_{sv}+\beta_{1t}T_{tv}+\beta_{2st}(S_{sv}*T_{tv})+\varepsilon_{bvst}$$

with residuals *ε*_*bvst*_. *P*-values less than 0.05 of the fixed regression coefficients were considered significant. In addition to the regression coefficients, the estimated marginal means of the model were used to describe the condensed gPDC differences of the switch conditions at both time points.

In order to assess if the observed changes in network characteristics are biased by simple stochastic or mechanistic effects that stem mainly from changes in very basic topological properties, we performed a surrogate-assisted analysis [36–38] using random networks obtained as described elsewhere [39], that were then compared to the functional networks (for details see S1 File) to help report changes in functional brain network characteristics more conclusively.

All analyses were performed with SAS 9.3 for Windows and R 3.2.0 for OSX.

## Results

**Fig 4:**
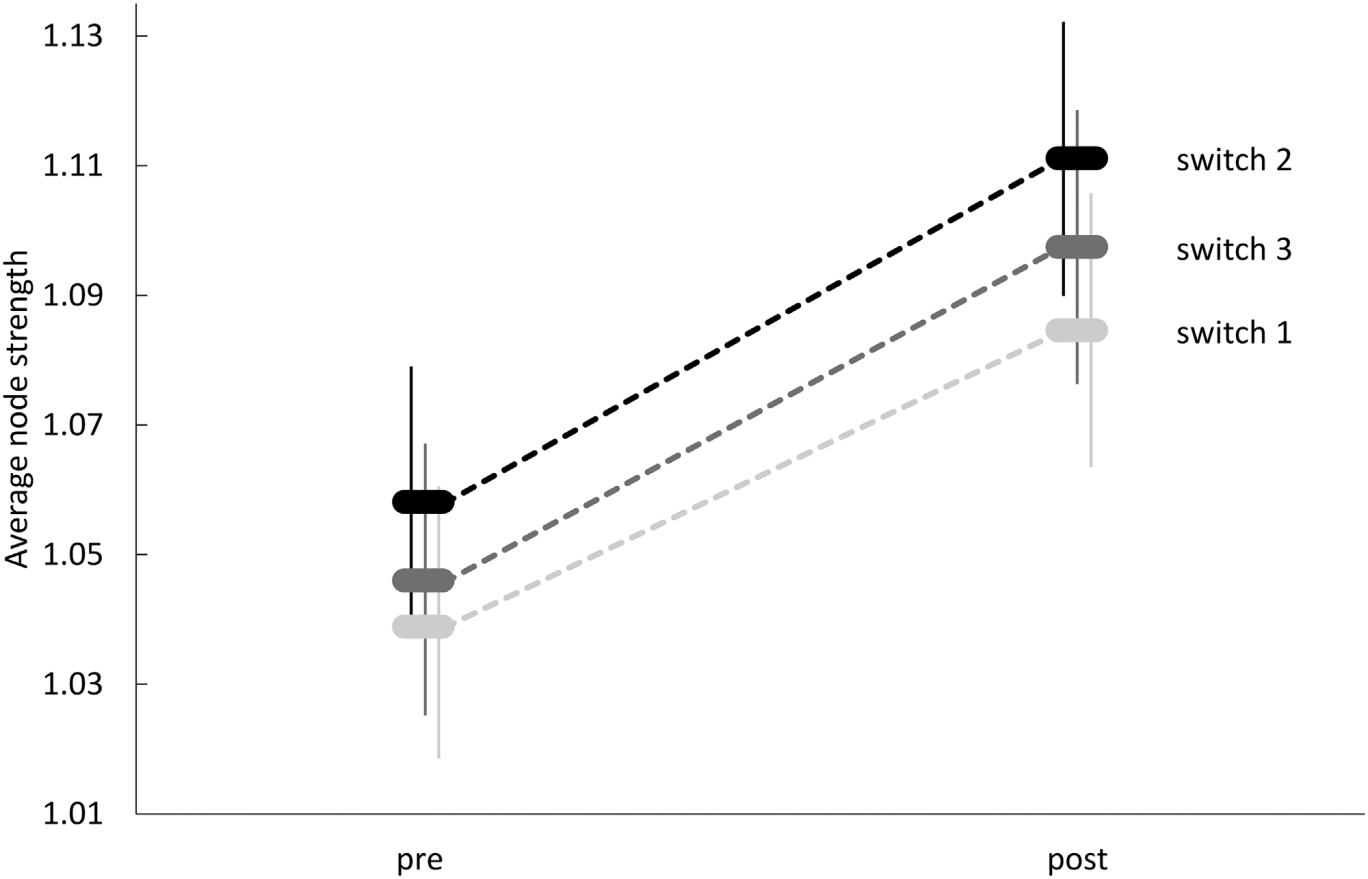
Linear mixed model estimates and standard errors of mean average node strength.

We found a global lithium treatment effect (*p* = 4.7·10^−7^), where the average node strength with respect to the aggregated gPDC-values was increased by lithium treatment. We did not find significant differences between switching conditions or interactions between treatment and switching conditions (Fig 4). The increased average node strength of the networks reflects a network-wide increase in strength of directed interactions between the nodes. A more detailed look at single frequency bins reveals a very homogenous image. In fact, we showed a significant increase of the average node strength between pre and post lithium therapy in a broad frequency window ranging from 0.015 Hz to 0.1 Hz with *p* < 10^−3^ for frequencies between 0.025 and 0.04 Hz, and *p* < 10^−4^ for frequencies between 0.04 and 0.1 Hz, respectively. The increase in interaction strength after lithium treatment constitutes the main finding of this study. We also found a global lithium effect on the characteristic path length (*p* < 10^−7^), which was decreased by the lithium treatment, and on all seven weighted clustering coefficients (*p* < 10^−4^), which were increased after treatment.

We also found statistical differences in weighted clustering between the functional brain networks (of either the pre lithium therapy condition or the post lithium therapy condition) and their randomized surrogate counterparts for some of the nodes (for details see S1 File).

Due to the absence of a control group we supplemented the LMM analysis by a permutation test, which randomizes the pre/post status label for each subject. Thus, a sample size of seven results in 128 = 2^7^ possible permutations. According to the LMM, the test statistic is the mean difference between post and pre treatment average node strength. Under the null hypothesis that the status label does not matter, we obtained a p-value of 0.031, which indicates a significant increase of the average node strength in a pre/post treatment comparison.

Local lithium effects measured by the in- or out-strength for each node, are presented in Table 2. The left side of the table depicts the regression coefficients *β*_1t_ (pre/post treatment status as fixed factor) and their standard errors for the LMMs with local out-degrees as dependent variable. For each ROI negative coefficients indicate a lower out-degree in the pre lithium treatment condition in comparison to the post treatment situation. The Benjamini–Hochberg procedure [40] was used to meet a 5% false discovery rate. Statistically significant regression coefficients were obtained for the ROIs marked in bold. The right putamen (ROI2), right orbital gyrus (ROI5), and the right subcallosal gyrus (ROI7) are regions with an increased out-degree post lithium treatment (intensified sources). Similarly, Table 2 right depicts the regression coefficients and their standard errors for the LMMs with local in-degrees as dependent variable. In this case, the right putamen (ROI2), right medial frontal gyrus (ROI3), left orbital gyrus (ROI4), and right lateral occipital cortex (ROI6) show significant increases in the local in-degree post lithium treatment (intensified sinks).

**Table 2:** Regression coefficients *β*_*1t*_, standard errors *SE* and p-values *p* for the LMM with the out-degree and in-degree as dependent variable (statistically significant regression coefficients in bold). For each ROI negative coefficients indicate lower local node strength in the pre lithium treatment condition in comparison to the post treatment situation.

	*Out-degree*		*In-degree*	
ROIs	*β*_*1t*_ *(SE)*	P-value	*β*_*1t*_ *(SE)*	P-value
ROI1-Right cerebellum	-0.033 (0.034)	0.333	-0.026(0.025)	0.308
ROI2-Right putamen	**-0.076 (0.029)**	**0.009**	**-0.068(0.026)**	**0.008**
ROI3-Right medial frontal gyrus	-0.011(0.037)	0.762	**-0.066(0.025)**	**0.008**
ROI4-Left orbital gyrus	0.023(0.045)	0.605	**-0.072(0.028)**	**0.009**
ROI5-Right orbital gyrus	**-0.073(0.028)**	**0.010**	-0.023(0.029)	0.413
ROI6-Right lateral occipital cortex	0.051(0.042)	0.228	**-0.071(0.025)**	**0.004**
ROI7-Right subcallosal gyrus	**-0.236(0.042)**	**<0.001**	-0.022(0.028)	0.430

**Fig 5:**
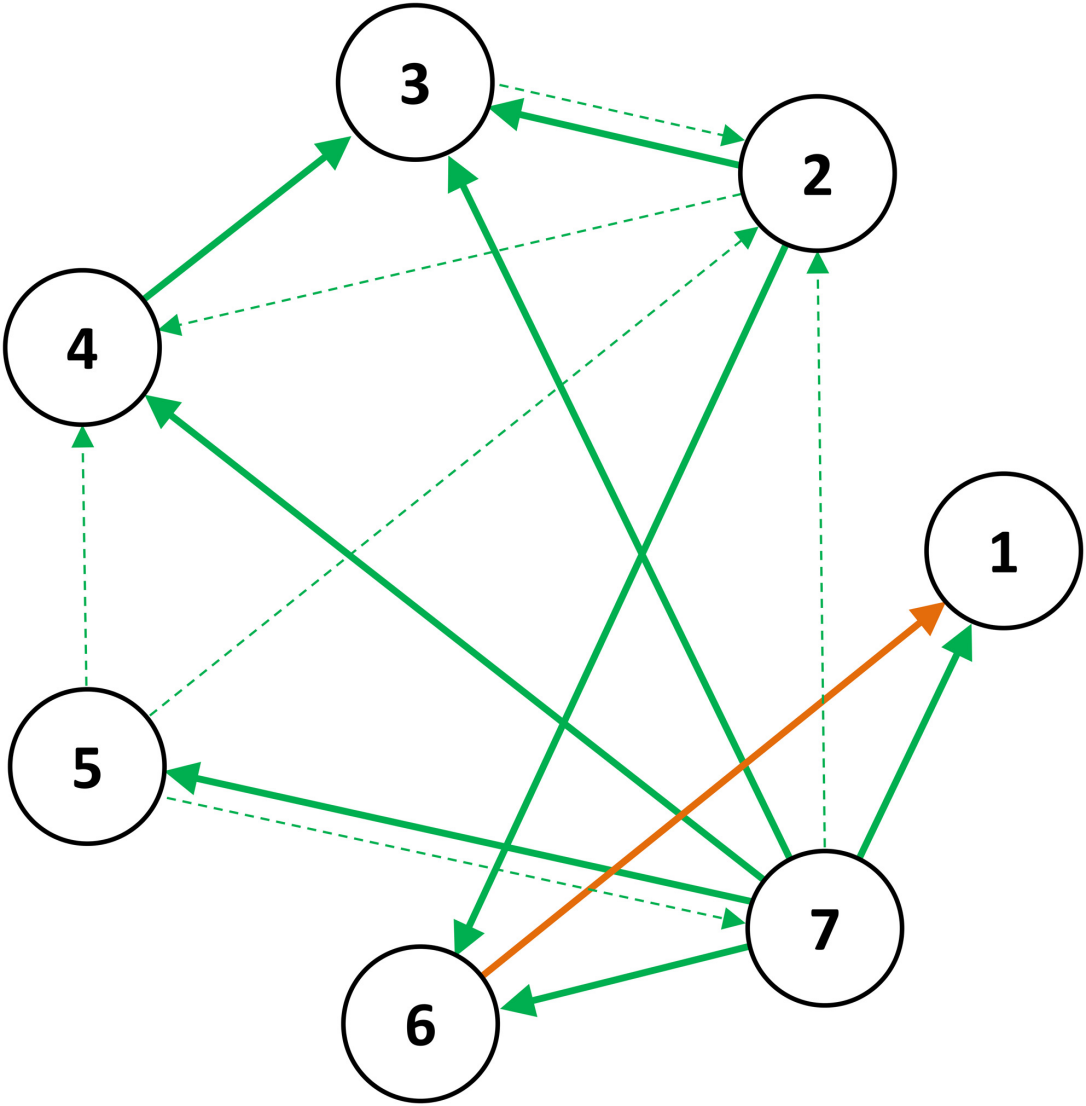
Edge weights significantly altered after lithium treatment: bold green—significant increase, bold orange—significant decrease. Additional edge weights with p-values less than 0.05 that were not significant after alpha adjustment, are represented in thin dashed arrows.

The finest analysis level is given by single edge weights defined by gPDC-values. Fig 5 shows edges with significantly altered weights after the lithium treatment (*α* = 0.05, adjusted for multiple comparisons according to Benjamini–Hochberg [40]). The results of this analysis are in line with results of all coarser analysis levels (local in- and out-strength, average node strength). The most prominent ROIs are ROI7 (right subcallosal gyrus) and ROI3 (right medial frontal gyrus). The right subcallosal gyrus exhibits many outgoing edges showing an increased aggregated gPDC after treatment. In contrast, the right medial frontal gyrus noticeably features many incoming edges with significantly increased edge weights post treatment. A similar, albeit weaker, finding may be attributed to ROI2 (right putamen) and ROI6 (right lateral occipital cortex). As shown in Table 2, the right subcallosal gyrus and the right putamen may be considered as intensified sources. The right medial frontal gyrus and the right lateral occipital cortex as intensified sinks have increased edge weights associated with interactions from the right subcallosal gyrus as well as the right putamen. The right orbital gyrus (ROI5, source), the right putamen (ROI2, sink), and the left orbital gyrus (ROI4, sink) are labeled as ROIs exhibiting significant gPDC changes in Table 2. Based on alpha-adjusted p-values (Fig 5, bold arrows) this summarized effect is not reflected at the level of single edge weights. In addition, we observed six additional edge weights involving ROI5 (source), ROI2 (sink), and ROI4 (sink), where the associated p-values are less than 0.05 (Fig 5, thin dashed arrows). However, after alpha adjustment, these alterations were not significant.

## Discussion

In this proof-of-concept study we combined information from structural and functional imaging data of cognitively impaired HIV-infected patients, and gPDC-based measures, to determine whether treatment-induced changes in brain function and microstructure are accompanied by changes in brain connectivity patterns.

Average node strength was increased by lithium treatment, which refers to a network-wide increase in strength of directed interactions between the nodes. In the context of HIV-associated cognitive impairment this may represent improvement in processing and forwarding of information in the underlying functional brain networks. In addition, when we considered other graph indices, in particular the characteristic path length and weighted clustering coefficients [31–33], the findings were similar to those we observed for the average node strength. These results are consistent and suggest that the global increase of interaction strengths is the major reason for changes in network indices. In our study this effect is emphasized by high correlations between average node strength and characteristic path length (correlation: -0.85), or clustering coefficients (correlation between 0.80 and 0.88), respectively. A decrease of the characteristic path length post treatment could be associated with the benefit of lithium treatment in restoring or building efficient connections for information transfer between nodes in the underlying brain network. This finding is in line with the alleged facilitated information transfer as seen in the average node strength of the networks, and might indicate that lithium treatment could improve neuronal plasticity as part of its effect on neurogenesis [6]. It remains in part unresolved to what extent the changes in the characteristic path length and weighted clustering coefficients are directly caused by increased edge weights post lithium treatment or if additional functionally relevant effects are at work, too. Although there is no single method to approach this issue, we addressed it by using a surrogate-assisted analysis [36–39] of these network characteristics; however, the results did not provide unequivocal answers.

To the best of our knowledge, gPDC has not been used before to show how lithium treatment affects functional connectivity. In particular it has never been shown that the interaction strengths are increased, which indicates a positive overall effect of lithium treatment on cognitive performance of HIV-infected patients with cognitive impairment.

In the present study we used weighted complete graphs. Other possibilities would have been to either threshold the weighted graphs so that they only contain weighted edges with an edge weight above the threshold or to threshold and dichotomize edges, which yields unweighted graphs. In this regard there is no completely satisfying solution for the problem of finding the right network type [10]. Based on our clinical data set, the concept of graph dichotomization based on statistical significance tests would not have been feasible due to disproportional high computational costs of Bootstrap approaches [28, 41], leaving systematic thresholding analyses as the only possible way to dichotomize weighted gPDC networks and to evaluate threshold-dependent graph characteristics. It is a generally used and accepted approach to analyze binary networks resulting from thresholding and dichotomizing the original weighted network or applying a multiple threshold strategy to a weighted network to yield a series of binary network instances over a wide range of thresholds [30]. These functions have to be additionally introduced in statistical analyses at the expense of additional post-processing steps or a statistical power reduction. Thus, in this study we considered weighted networks directly in order to circumvent information loss.

The temporal selectivity of tvMVAR models addresses the dynamic behavior of brain response in the course of the experiment. Frequency selectivity of gPDC enables an integration of the task sequence by using the spectral properties of the experimental protocol. Nevertheless, a drawback of this analytical approach is the high number of processing steps and the accompanying possibility of making wrong decisions in each of these steps.

Another interesting aspect in a pre-post treatment comparison of functional connectivity measures are the so called hidden sources. In general they represent a problem in connectivity analyses even if multivariate approaches are applied, because the existence of latent variables may result in spurious interactions. Eichler [42] developed a graphical method that enables the determination of causal relations in the presence of possible spurious connections. His method is particularly interesting when graphs with a small number of nodes have to be identified, because an exhaustive analysis of many subseries of the full time series has to be performed. Thus, an obvious drawback of this graphical approach is that statistical errors, related to multistep autoregressive model fits, avoid a comparison of different graphical representations, which would be necessary to investigate therapy effects. In our study, we do not assume that our ROI set is free from latent variables with the consequence of possible spurious interactions. If latent variables remain unchanged during the treatment, the observed lithium effect is then directly caused by the considered ROIs. If latent variables change during treatment, these alterations may be indirectly mirrored by the analyzed network, regardless of direct changes in the functional connectivity between the considered ROIs. Thus, we still observe a lithium effect on functional connectivity but the specific localization remains potentially concealed.

A limitation of the study is the small number of data sets available for analysis and the lack of an appropriate control group due to the open-label nature of the study. Due to the small number of subjects and the short duration of the study, our previous work demonstrated only a trend toward improvement in cognitive performance and no significant clinical changes [8], thus making it difficult to relate imaging outcomes to these changes. It is of interest that the orbitofrontal and mediofrontal cortices as well as the putamen were among the ROIs where lithium increased the local node strength, as these areas have previously been reported to be affected in HIV–associated CNS injury [43, 44].

Our findings suggest that lithium treatment of HIV infected individuals induces changes in brain microstructure (as assessed by DTI) that are associated with improved performance related features of brain functional network connectivity (as assessed by fMRI). The increase in interaction strength after lithium treatment suggests a positive effect of lithium treatment on functional connectivity in HIV-infected patients, which to the best of our knowledge has never been reported before. The use of gPDC-based measures provides insights into brain connectivity by revealing underlying interactions, their strength and direction. Therefore, gPDC-based measures offer an advanced way of understanding changes in functional and anatomical interrelations of brain networks in the context of a disease process and in response to pharmacological intervention.

## Supporting Information

S1 File Surrogate network analysis. (DOCX)
