# Supplementary material for: Are Structural Changes Induced by Lithium in the HIV Brain Accompanied by Changes in Functional Connectivity?
Source: PLoS One. 2015 Oct 5;10(10):e0139118. doi: 10.1371/journal.pone.0139118 (PMC4593570; doi:10.1371/journal.pone.0139118)
Supplement: S1 File — (DOCX) [file pone.0139118.s001.docx]

**Surrogate-network analysis**

**Methods**

In order to assess if the observed changes in network characteristics are biased by simple stochastic or mechanistic effects that stem mainly from changes in very basic topological properties, we performed a surrogate-assisted analysis [1-3]. For each functional brain network the observed network characteristics were normalized by dividing them by their mean value in a sample of 1000 surrogate networks with randomized topologies (null model). As surrogates we used random networks that preserve the edge weights of the weighted complete functional brain network by means of global edge weight permutations [4]. Statistical differences between network characteristics in functional brain networks and their average values in the surrogate network sample were identified with the paired two-sided Wilcoxon signed rank test at a significance level of 0.05. This surrogate-assisted analysis approach helps to report changes in functional brain network characteristics more conclusively.

**Results**

*Weighted clustering coefficients*. We found statistical differences between the functional brain networks (of either the pre lithium therapy condition or the post lithium therapy condition) and their randomized surrogate counterparts.

*Pre-lithium treatment condition*.

Weighted clustering coefficients of the right medial frontal gyrus (ROI3, p<10^-16^), left orbital gyrus (ROI4, p = 0.009) and right lateral occipital cortex (ROI6, p<10^-16^) were larger in the functional brain networks than in the surrogate networks. For these nodes the median values (and interquartile range) of the normalized weighted clustering coefficients were 1.087 (0.176), 1.028 (0.181) and 1.074 (0.151), respectively. There were no statistical differences between the real and surrogate network weighted clustering coefficient of the right subcallosal gyrus (ROI7, p = 0.301). The median value (and interquartile range) of the normalized weighted clustering coefficients was 1.006 (0.139). The weighted clustering coefficients of right cerebellum (ROI1, p=1.93x10^-9^), right putamen (ROI2, p=1.83x10^-6^) and right orbital gyrus (ROI5, p=6.77x10^-10^) were smaller in the functional brain networks than in the surrogate networks. For these nodes the median values (and interquartile range) of the normalized weighted clustering coefficients were 0.943 (0.166), 0.969 (0.129) and 0.952 (0.131), respectively.

*Post-lithium treatment condition*.

Weighted clustering coefficients of the right medial frontal gyrus (ROI3, p<10^-16^), right lateral occipital cortex (ROI6, p=1.33x10^-12^) and right subcallosal gyrus (ROI7, p<10^-16^) were larger in the functional brain networks than in the surrogate networks. For these nodes the median values (and interquartile range) of the normalized weighted clustering coefficients were 1.070 (0.142), 1.062 (0.163) and 1.060 (0.157), respectively. There were no statistical differences between the real and surrogate network weighted clustering coefficient of the right putamen (ROI2, p = 0.868) and the left orbital gyrus (ROI4, p = 0.985). The median values (and interquartile range) of the normalized weighted clustering coefficients were 0.997 (0.145) and 0.998 (0.195), respectively. The weighted clustering coefficients of the right cerebellum (ROI1, p<10^-16^) and the right orbital gyrus (ROI5, p=4.46x10^-6^) were smaller in the functional brain networks than in the surrogate networks. For these nodes the median values (and interquartile range) of the normalized weighted clustering coefficients were 0.914 (0.164) and 0.967 (0.165), respectively.

To summarize, the weighted clustering coefficients of all nodes were increased post lithium treatment and for at least some of the nodes we cannot entirely rule out that this increase can be explained, at least in part, by nontrivial local changes in the underlying networks. However, since the effect sizes were small we refrain from emphasizing the increase in clustering in our interpretation of the results, and focus on the increase in interaction strength after lithium treatment.

Contrary to the situation for the weighted clustering coefficient, the value of the characteristic path length is not changed in the surrogate networks. The reason for this is that the functional brain networks are complete, which means that every node is connected to all other nodes in the network and the edge weights and the connection lengths (costs) obtained by a transformation of the edge weights [5] are homogenous enough so that the direct connections between node pairs are always the shortest ones. Since the collection of edge weights is preserved in the surrogate networks, the characteristic path length does not change.

References

1. Ansmann G, Lehnertz K (2012) Surrogate-assisted analysis of weighted functional brain networks. J Neurosci Methods 208: 165-172.

2. van den Heuvel MP, Sporns O (2011) Rich-club organization of the human connectome. J Neurosci 31: 15775-15786.

3. Humphries MD, Gurney K (2008) Network 'small-world-ness': a quantitative method for determining canonical network equivalence. PLoS One 3: e0002051.

4. Barrat A, Barthelemy M, Pastor-Satorras R, Vespignani A (2004) The architecture of complex weighted networks. Proceedings of the National Academy of Sciences of the United States of America 101: 3747-3752.

5. Mantegna RN (1999) Hierarchical structure in financial markets. The European Physical Journal B-Condensed Matter and Complex Systems 11: 193-197.
